# Basal levels of inorganic elements, genetic damages, and hematological values in captive *Falco peregrinus*

**DOI:** 10.1590/1678-4685-GMB-2022-0067

**Published:** 2022-05-27

**Authors:** Julian Stocker, Ana Paula Morel, Micaele Wolfarth, Johnny Ferraz Dias, Liana Appel Boufleur Niekraszewicz, Cristina V. Cademartori, Fernanda R. da Silva

**Affiliations:** 1 Universidade La Salle, Laboratório de Ecogenotoxicologia, Canoas, RS, Brazil.; 2 Universidade La Salle, Programa de Pós-graduação em Avaliação de Impactos Ambientais, Canoas, RS, Brazil.; 3 Universidade Federal de Pelotas (UFPEL), Programa de Pós-Graduação em Clínica Médica Veterinária, Pelotas, RS, Brazil.; 4 Universidade Federal do Rio Grande do Sul (UFRGS), Laboratório de Implantação Iônica, Instituto de Física, Porto Alegre, RS, Brazil.; 5 Universidade La Salle, Programa de Pós-graduação em Memória Social e Bens Culturais, Canoas, RS, Brazil.; 6 Universidade La Salle, Programa de Pós-graduação em Saúde e Desenvolvimento Humano, Canoas, RS, Brazil.

**Keywords:** Biological monitoring, basal DNA damage, falcon, reference value, hematology

## Abstract

It is essential to determine the basal pattern of different biomarkers for future evaluation of animal health and biomonitoring studies. Due to their great displacement capacity and to being at the top of their food chains, birds of prey are suitable for monitoring purposes. Furthermore, some birds of prey are adapted to using resources in urban places, providing information about this environment. Thus, this study determined the basal frequency of micronuclei and other nuclear alterations in peripheral blood erythrocytes of *Falco peregrinus*. Hematological and inorganic elements analysis were also performed. For this purpose, 13 individuals (7 females and 6 males) were sampled in private breeding grounds. Micronucleus, nuclear buds, nucleoplasmic bridges, notched nuclei, binucleated cells and nuclear tails were quantified. Inorganic elements detected included the macro-elements Ca, P, Mg, Na, Cl, S and K as well as the micro-elements Fe, Al and Zn. Our study found similar values compared to previous studies determining the reference ranges of hematologic parameters in falcons. The only different value was observed in the relative number of monocytes. Thus, this study is the first approach to obtaining reference values of cytogenetic damage in this species and could be useful for future comparisons in biomonitoring studies.

In recent years, different organisms have provided information about environmental quality (Parmar et al., 2016). Birds of prey due to their great displacement capacity and to being at the top of the food chains are suited for monitoring purposes (Lodenius and Solonen, 2013). Top bird predators have been evaluated regarding exposure to pesticide, metal and other chemical substances (Lodenius and Solonen, 2013; de Wit et al., 2019; Aver et al., 2020; Frixione and Rodríguez-Estrella, 2020). In biomonitoring studies, it is possible to evaluate biomarkers at the molecular, cellular, morphological and physiological levels in different species. Among the available bioassays, there are several that are more invasive or less invasive to animals (Valdes, 2010). A technique that estimates the exposure level in DNA without having to sacrifice the organism exposed is the Erythrocyte Nuclear Abnormalities (ENA) assay. The ENA assay is considered a proper tool for detecting the DNA damage because its analysis includes micronuclei and the other nuclear alterations analyses (Gomes et al., 2015). 

Most studies about genotoxicity in birds include only micronuclei in analysis, excluding the other nuclear variations, which may be more frequent than micronuclei. Nuclei with smaller or larger evaginations, nuclei with vacuoles and nuclei with a deep slit are alterations that can also be observed (Carrasco et al., 1990; Quero et al., 2016; de Souza et al., 2017; de Faria et al., 2018; Silveira et al., 2022).

Some birds of prey, such as *Falco peregrinus*, have adapted to using resources in urban areas. According to Pollack et al. (2017) these species provide information, especially about the widespread consequences of urbanization, giving an insight into its influence on animal behavior and physiology, as well as guiding investigations in humans. 

The aim of this study was to determine the frequency of micronuclei and other nuclear alterations in peripheral blood erythrocytes of *Falco peregrinus*, as a first approach to obtain reference values of cytogenetic damage in this species. Furthermore, knowledge was obtained regarding other physiological parameters, such as hematological analysis and the quantification of inorganic elements.

All birds used in this study belong to Criatório Hayabusa - Consultoria Ambiental Ltda., a wildlife center and commercial breeder located in São Francisco de Paula (Rio Grande do Sul, Brazil). The area has approximately 48 ha and is considered to be in an excellent state of conservation, distant from urban areas and areas where crops are cultivated. The individuals are housed in outdoor aviary cages (4 m (width) x 4 m (height) x 4 m (length)) containing a couple of birds each. The birds had been captive for at least four years and were fed daily with *Coturnix coturnix* and water *ad libitum.* The sex of the birds was determined through external sexual size dimorphism, wherein the male can carry up to 50% less loads than the female (Mills et al., 2019). In addition, the bird’s age (juvenile/adult) was defined according to information on a metal ring.

The procedures involving animals were conducted in compliance with the guidelines approved by the Committee on Ethics in the Use of Animals of the Universidade La Salle (CEUA-UNILASALLE, number 003/2017), and authorized by the Ministry of the Environment (MMA) through the Sistema de Autorização e Informação da Biodiversidade (SISBIO) for scientific activities (number 59921-1).

Blood samples of 13 individuals, collected by the center’s veterinarian, were drawn from the ulnar vein of the wing using heparinised syringes. The samples were immediately smeared onto clean glass slides, where two slides were prepared per individual. The slides were sent to the laboratory. Remaining blood samples were also transported to the laboratories at below 8 ºC for the analysis of hematological and inorganic elements. The animals were identified as to sex and age (juvenile/adult). All the birds sampled were apparently healthy, without any signs of illness.

In the laboratory, the slides were prepared according to Grisolia and Cordeiro (2000). At least 2,000 erythrocytes for each animal were scored using bright-field optical microscopy at a magnification of ×200-1000. Coded slides were blind-scored by a single observer. The presence of ENA was evaluated according to procedures by Carrasco et al. (1990) and Quero et al. (2016), using mature erythrocytes to estimate the frequency of the following nuclear lesions: (i) micronuclei (MN); (ii) nuclear buds (NBud); (iii) binucleated cell (BN); (iv) nuclear tails (NT); (v) nucleoplasmic (NB); and (vi) Notched (NO).

The content of inorganic elements in the blood samples was analyzed by particle-induced X-ray emission (PIXE) (Johansson et al., 1995). As the PIXE system requires the use of solid samples, the blood samples were dried at 60 °C. Once dried, the samples were homogenized and pressed into 2 mm thick pellets before being placed in the target holder inside the reaction chamber (pressure about 10^-5^ mbar). A 3 MV Tandetron accelerator provided a 2.0 MeV proton beam with an average current of 3 nA at the target. The X-rays produced in the samples were detected by a Si(Li) detector with an energy resolution of ca. 150 eV at 5.9 keV. The spectra were analyzed with the GUPIX software package and the results were expressed in mg/g (Campbell et al., 2000). The same sample was evaluated in three independent analyses in order to obtain mean and standard deviation.

The hematological evaluation was carried out in a commercial laboratory (BLUT’S Centro de Diagnóstico, Produtos e Serviços Veterinários, Porto Alegre-RS, Brazil) according to standard methods. Together with the results of the present study, information about other studies were taken as reference to interpret hematologic parameters.

The normality of the variables was evaluated using the Kolmogorov-Smirnov test. To compare the parameters of the study population, Student*t*-, and Mann-Whitney U non-parametric tests were used. The critical level for rejection of the null hypothesis was considered to be P < 0.05.

The interspecific variations in the spontaneous frequencies of nuclear alterations are probably related to the intrinsic individual factors associated with ingestion, accumulation, metabolism and excretion of the xenobiotics to which the organism is exposed daily. Furthermore, the correct functioning of the DNA repair also could be involved in this interspecific response to DNA damage (Jha, 2004). 

Information on 13 animals sampled is presented in Table 1. It this study 7 females and 6 males were collected, all adults, where no significant difference between the bird sex was observed when the erythrocyte nuclear abnormality (ENA) frequencies were compared (P>0.05). Micronucleus, nuclear buds, nucleoplasmic bridges, notched nuclei, binucleated cells and nuclear tails were the ENAs observed in *F. peregrinus* cells (Table 1). Nuclear alterations with a higher frequency were nuclear buds. Nucleoplasmic bridges were observed only in female specimens.

**Table 1 -:** Sample size and frequency of nuclear abnormalities in erythrocytes (2,000 cells) of *Falco peregrinus.*

	Female (n=7)	Male (n=6)	Total (n=13)
Micronucleus	1.29 ± 1.50	2.00 ± 1.26	1.62 ± 1.39
Nuclear buds	3.14 ± 2.34	1.5 ± 1.52	2.38 ± 2.10
Nucleoplasmic bridges	0.43 ± 0.53	0	0.23 ± 0.44
Nuclear tails	1.14 ± 1.07	0.5 ± 0.55	0.85 ± 0.90
Notched nuclei	0.14 ± 0.38	0.33 ± 0.82	0.23 ± 0.60
Binucleated cells	1.29 ± 1.38	1.17 ± 1.94	1.23 ± 1.59

Mann-Whitney test to compare female and male. Data expressed in mean ± standard deviation.

According to Zúñiga-González et al. (2001), species with the highest values of MNs basal frequency potentially could be useful for biomonitoring the possible effect of environmental mutagens. Birds of prey are sensitive indicators of environmental quality because they are particularly prone to bioaccumulate organic contaminants (Carneiro et al., 2016), including genotoxins. However, there is scarce information concerning spontaneous MN frequency in birds, mainly in birds of prey. In our study, the mean MN frequency was 0.8 per 1,000 erythrocytes analysed, values ​​higher than determined for *Buteo albicaudatus* and *Polyborus plancus,* that are other species of birds of prey of the Falconidae family. No micronucleus was found in *Polyborus plancus* while the rate for *Buteo albicaudatus* was 0.05 micronuclei (Zúñiga-González *et al.,* 2001). In another study, Zúñiga-González *et al.* (2000) evaluated spontaneous micronuclei in birds of prey and did not observe MN in *Accipiter cooperi*, *Polyborus plancus*, *Aquila chrysaetos*, and *Parabuteo unicinctus.* For *Falco sp.* and *Buteo sp.* the MN frequencies were 0.14 and 0.02 respectively. 

Regarding other abnormalities evaluated by ENA assay, a rate of 1.19 NBud per 1,000 erythrocytes was counted. NBud reflects chromosomal instability and it is related to DNA amplification, DNA repair complexes and excess chromosomes due to aneuploid events (Fenech et al., 2011). There is no information concerning ENA frequencies in birds of prey. In birds, Quero et al. (2016), evaluating 17 different species of wild birds, observed that 80.9 % of the individuals presented at least one NBud with a rate of 0.10 to 0.95 ± 0.14/1000 erythrocytes. A high frequency of NBud (1.28/1000 cells) was observed in individuals of *Aratinga canicularis* exposed to water (negative control) (Gomez-Meda et al., 2006). This study evaluated the MN and NBud frequencies in birds exposed to mitomycin-C, suggesting that budding may reflect a wider spectrum of DNA damage than the MN formation. Thus, the authors proposed that estimating the NBud rate in routine hematological analysis could serve to establish basal values for the species and to evaluate environmental genotoxicity exposure.

In our study, the individuals also presented NB and NT in their erythrocytes. Molecularly, these nuclear alterations could be formed by the same pathway (Anbumani and Mohankumar, 2015). When there is dicentric chromosome formation due to misrepair of DNA breaks, telomere end fusions or incorrect sister chromatid separation, this chromosome can be pulled to opposite poles of the cell during mitosis, producing the nucleoplasmic bridges (Fenech et al., 2011). It is also possible that a cytoplasmic constriction of the NB could result in a nuclear tail (Anbumani and Mohankumar, 2015). *Falco peregrinus* showed a rate of 0.12 nucleoplasmic bridge and 0.43 nuclear tails for 1000 erythrocytes. There are no previous studies including frequencies of these nuclear alterations in birds of prey. However, Quero et al. (2016) in different orders found a mean range between 0.01 and 0.20 for NB as well as 0.05 and 0.22 for NT in peripheral blood erythrocytes.

In addition, the evaluation of NO cells in this work showed a mean frequency of 0.12/1000 erythrocytes. The mechanisms responsible for NO cell formation must be better understood, although de Faria et al. (2018) comment on nuclei with asymmetric constriction such as notched type that can be associated with damage in structures/proteins that leads to the cleavage of different nuclear and cytoskeleton proteins and to tubulin polymerization failure, for example. Quero et al. (2016) found mean nuclear alterations between 0.1 and 2.5 in birds analyzed, with results similar to those reported here.

Erythrocytes with two nuclei present cytokinesis failure. BN cell formation is related to erroneous mitosis, where karyokinesis is not synchronized with cytokinesis (Coonen et al., 2004). In *F. peregrinus* the BN cell rate was 0.62/1000 cells. Regarding BN cells in birds, reported mean frequencies were between 0.05 and 0.40 for bird species (Quero et al., 2016).

As to age and sex influence on spontaneous nuclear alterations, Shepherd and Somers (2012) have shown that these are important factors affecting background MN frequency. In their study, male birds had a 1.4- to 2.2-fold higher frequency of MN than females. In our research, all birds tested were adults and with regard to sex, NB cells were observed only in female individuals. Other authors showed that the sex of the birds did not affect nuclear alteration in the control group (de Souza et al., 2017).

Inorganic elements detected in the blood of birds of prey included the macro-elements Ca, P, Mg, Na, Cl, S and K as well as the micro-elements Fe, Al and Zn (Figure 1). In Figure 1A it appeared that Na (4.25 mg/g) was the highest concentration, while Zn (0.018 mg/g) was the lowest element (Figure 1B). No difference was observed between male and female (P>0.05). 

The analysis of elements, mainly metals in birds, has been an important tool to assess environmental pollution because human activities have increased the natural environment concentrations (Carneiro et al., 2016). In our study, the basal quantity of some macro and micronutrients was detected in the blood of captive birds of prey not exposed to contaminants. Carneiro *et al.* (2016), in a review about biomonitoring of metals and metalloids using raptors in Portugal and Spain, point out that the blood and liver samples were very frequently used the studies. 

**Figure 1 -:**
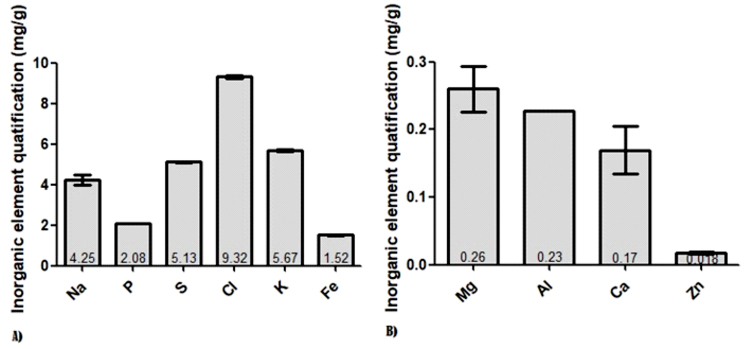
A and B) Levels of inorganic elements in blood samples of *Falco peregrinus*. Data expressed in mg/g, mean ± standard deviation.

Metals, such as Fe and Zn, were detected in bird blood in our study. Fe is needed in the hemoglobin production for the blood to carry oxygen. However, as the Fe homeostasis must be maintained, little iron in the diet can cause anemia in birds and too much can lead to iron storage disease (Cork, 2000). Zn is also an important micronutrient with physiologic benefits, including bone formation, immune function and normal functioning of the central nervous system. In birds, overexposure to zinc can result in anemia (Puschner and Poppenga, 2009). In our study, the findings indicate the presence of Al in the blood of birds of prey. The origin of Al found in the birds is as yet unknown due to an increase in the redistribution of this element in the environment as a result of human activities. This element is abundant in the Earth’s crust and is moved by natural and/or human activity. Atmospheric precipitation may be acidic, enhancing the Al leaching. However, levels of A1 below 0.1% generally do not have an adverse effect on the overall health of the animal, but higher levels may cause decreased growth rates and muscle weakness (Scheuhammer, 1987).

Results of hematological values are summarized in Table 2. When it comes to bird sex, there was significant variation (P<0.05) of the hematological values. In males, the values of relative numbers of basophils (1.50±0.71) were significantly higher than the values for females (0.17±0.41). Previous studies determining the reference ranges of hematologic parameters in falcons found similar values to our study, although the number of healthy birds in this study (n = 13) was smaller than those included in reports by Wernery et al. (2004) (n = 267), Muller et al. (2005) (n = 320) and Padrtova and Lloyd (2009) (n = 96). A different value was observed in the relative number of monocytes, where in the cited studies they range from 1.8 to 4.4, while in our study it was 10.75±4.81. In birds, monocyte cells can be confused with lymphocytes during cell count, and variations the results may reflect this difficulty (Ivins et al., 1986).

**Table 2 -:** Hematological values for *Falco peregrinus* and reference ranges of falcons from the literature.

	Present study	Reference 1	Reference 2	Reference 3
Red blood cells (x10^6^/uL)	2.88 ± 0.36	2.68 ± 0.23	3.35 ± 0.12	2.39 ± 0.26
Hemoglobin (g/dL)	15.75 ± 1.48	17.9 ± 1.1	15.3 ± 0.6	17.9 ± 1.6
Hematocrit (%)	49.00 ± 5.29	N.D.	46 ± 2	N.D.
MCV (fL)	172.99 ± 28.52	176.5 ± 10.3	137.3 ± 4.2	219.7 ± 25.4
MCHC (%)	32.18 ± 28.52	38.1 ± 2.0	N.D.	34.4 ± 1.5
Leukocytes (x10^3^/uL)	5.44 ± 1.21	5.63 ± 1.63	9.31 ± 3.24	7.55 ± 2.27
Platelets	21.17 ± 6.96	N.D.	N.D.	N.D.
PP (g/L)	44.5 ± 6.22	N.D.	N.D.	N.D.
Heterophils (%)	66.75 ± 11.17	69.9 ± 10.0	60.0 ± 10.0	49.9 ± 3.5
Lymphocytes (%)	20.75 ± 7.92	25.3 ± 9.2	37.4 ± 11.2	44.2 ± 3.4
Monocytes (%)	10.75 ± 4.81	1.8 ± 1.6	2.6 ± 1.2	4.4 ± 1.6
Eosinophils (%)	1.25 ± 1.71	1.9 ± 1.9	0	1.4 ± 1.1
Basophils (%)	0.5 ± 0.71	1.1 ± 1.2	0	0.1 ± 0

MCV = Mean Corpuscular Volume; MCHC = Mean Corpuscular Hemoglobin Concentration; PP = Plasm Protein; Reference 1: Padrtova and Lloyd, 2009; Reference 2: Wernery et al 2004; Reference 3: Muller et al, 2005. N.D: Not determined.

In addition, changes were observed in hematological parameters between birds of prey of different sex, where the relative numbers of basophils were higher in males. Oliveira et al. (2014), comparing males and females of *Harpia harpyja*, found some variation in the values of relative number of basophils, with values increased in females. According to Campbell (1994) the function of basophils in poultry is not fully elucidated.

In order to evaluate environmental pollution using biomonitoring, it is essential to know the physiological parameters of the species studied, such as basal DNA damage, inorganic elements, and hematological values. In the present study, data on baseline MN and ENA frequencies for *Falco peregrinus* were first reported on captive species. Micronucleus, nuclear buds, nucleoplasmic bridges, notched nuclei, binucleated cells and nuclear tails were observed. Inorganic elements were also detected in the blood of bird of prey including macro and micro-elements as well, as hematological values. Thus, the data published in this study could be useful for future comparisons in biomonitoring studies and it can help the veterinarian in laboratory and clinical assessments of falcons kept in captivity in conservation programs.
